# Supplementary material for: The effects of mycorrhizal colonization on phytophagous insects and their natural enemies in soybean fields
Source: PLoS One. 2021 Sep 22;16(9):e0257712. doi: 10.1371/journal.pone.0257712 (PMC8457447; doi:10.1371/journal.pone.0257712)
Supplement: S1 Table — P <0.05; n = 48. (DOCX) [file pone.0257712.s001.docx]

**S1 Table.** Arbuscular mycorrhizal fungi root colonization and yield of soybean at Varennes and Saint-Simon based on inoculation treatments (Control (C), Mycorrhizae+Rhizobium (MR), Mycorrhizae+Rhizobium+Bacillus (MRB)), potassium treatments (K-: without potassium; K+: with potassium), tested individually and in interaction (*F-value, df, P-value*). *P* <0.05; n=48.

| Site | Plant parameters | Inoculants | | | Potassium | | | Inoculants:Potassium | | |
| --- | --- | --- | --- | --- | --- | --- | --- | --- | --- | --- |
|  |  | *df* | *F* | *P* | *df* | *F* | *P* | *df* | *F* | *P* |
| Varennes | AMF root colonization (%) | 2 | 0.66 | 0.53 | 1 | 0.18 | 0.67 | 2 | 0.62 | 0.54 |
|  | Yield (kg/ha) | 2 | 0.52 | 0.59 | 1 | 2.75 | 0.11 | 2 | 0.04 | 0.95 |
| Saint-Simon | AMF root colonization (%) | 2 | 0.28 | 0.75 | 1 | 4.26 | 0.06 | 2 | 0.24 | 0.79 |
|  | Yield (kg/ha) | 2 | 0.62 | 0.55 | 1 | 0.04 | 0.85 | 2 | 0.62 | 0.55 |
