# Supplementary material for: The effects of mycorrhizal colonization on phytophagous insects and their natural enemies in soybean fields
Source: PLoS One. 2021 Sep 22;16(9):e0257712. doi: 10.1371/journal.pone.0257712 (PMC8457447; doi:10.1371/journal.pone.0257712)
Supplement: S2 Table — Linear mixed effect model (LMM) follows by ANOVA. *: P <0.05; **: P <0.001; n = 48. (DOCX) [file pone.0257712.s002.docx]

**S2 Table.** Abundance of insect functional groups sampled on soybean at Varennes and Saint-Simon based on inoculation treatments (Control (C), Mycorrhizae+Rhizobium (MR), Mycorrhizae+Rhizobium+Bacillus (MRB)), potassium treatments (K-: without potassium; K+: with potassium), tested individually and in interaction (*F-value, df, P-value*). ^*^: *P* <0.05; ^**^: *P* <0.001; n=48.

| Site | Variables | Inoculants | | | Potassium | | | Inoculants:Potassium | | |
| --- | --- | --- | --- | --- | --- | --- | --- | --- | --- | --- |
|  |  | *df* | F | P | *df* | F | P | *df* | F | P |
| Varennes | Piercing-sucking insects | 2 | 4.12 | 0.026^*^ | 1 | 0.004 | 0.953 | 2 | 0.23 | 0.794 |
|  | Chewing insects | 2 | 0.26 | 0.770 | 1 | 1.55 | 0.253 | 2 | 0.30 | 0.744 |
|  | Aphids without *A.glycines* | 2 | 2.74 | 0.081 | 1 | 0.08 | 0.787 | 2 | 0.77 | 0.471 |
|  | Aphids natural enemies | 2 | 0.04 | 0.958 | 1 | 0.48 | 0.509 | 2 | 0.34 | 0.713 |
|  | *A. glycines* | 2 | 2.38 | 0.117 | 1 | 0.51 | 0.481 | 2 | 0.55 | 0.581 |
|  | *Empoasca* spp. | 2 | 1.88 | 0.176 | 1 | 0.99 | 0.330 | 2 | 0.04 | 0.959 |
|  | Shannon index of phytophagous insects | 2 | 0.66 | 0.510 | 1 | 0.13 | 0.723 | 2 | 0.24 | 0.783 |
| Saint-Simon | Piercing-sucking insects | 2 | 1.05 | 0.373 | 1 | 0.008 | 0.931 | 2 | 0.81 | 0.464 |
|  | Chewing insect | 2 | 0.14 | 0.870 | 1 | 0.004 | 0.951 | 2 | 0.98 | 0.388 |
|  | Aphids without *A.glycines* | 2 | 3.29 | 0.067 | 1 | 0.08 | 0.780 | 2 | 3.29 | 0.07 |
|  | Aphids natural enemies | 2 | 0.82 | 0.459 | 1 | 0.06 | 0.806 | 2 | 0.21 | 0.811 |
|  | *A. glycines* | 2 | 1.94 | 0.179 | 1 | 2.32 | 0.142 | 2 | 6.70 | 0.006^**^ |
|  | *Empoasca* spp. | 2 | 0.85 | 0.434 | 1 | 5.53 | 0.024^*^ | 2 | 0.23 | 0.792 |
|  | Shannon index of phytophagous insects | 2 | 0.57 | 0.570 | 1 | 1.30 | 0.265 | 2 | 0.015 | 0.984 |
