# Supplementary material for: The effects of mycorrhizal colonization on phytophagous insects and their natural enemies in soybean fields
Source: PLoS One. 2021 Sep 22;16(9):e0257712. doi: 10.1371/journal.pone.0257712 (PMC8457447; doi:10.1371/journal.pone.0257712)
Supplement: S3 Table — Values represent mean ± SE of 8 replicates (n = 48) in each site. Letters follow by mean ± SE indicate significant differences among treatments based on the Tukey’s honest significant difference (HSD) test after Linear mixed effect model (LMM) follow by ANOVA. *: P <0.05. (DOCX) [file pone.0257712.s003.docx]

**S3 Table.** Abundance of piercing-sucking insects and *Empoasca* spp. sampled on soybean at Varennes and Saint-Simon respectively based on inoculation treatments (Control (C), Mycorrhizae+Rhizobium (MR), Mycorrhizae+Rhizobium+Bacillus (MRB)) irrespective of potassium (K-: without potassium; K+: with potassium) and vice-versa. Values represent mean ± SE of 8 replicates (n=48) in each site. Letters follow by mean ± SE indicate significant differences among treatments based on the Tukey’s honest significant difference (HSD) test after Linear mixed effect model (LMM) follow by ANOVA. ^*^: *P* <0.05.

| Site | Variables | Potassium | | *F* | *df* | *P* | Inoculants | | | *F* | *df* | *P* |
| --- | --- | --- | --- | --- | --- | --- | --- | --- | --- | --- | --- | --- |
|  |  | K- | K+ |  |  |  | C | MR | MRB |  |  |  |
| Varennes | Piercing-sucking insects | 22.5±1.96 | 22.7±1.77 | 0.004 | 1 | 0.953 | 18.9±2.23a | 22.8±1.69 ab | 26.1±2.54 b | 4.12 | 2 | 0.026* |
| Saint-Simon | *Empoasca* spp. | 1.63±0.44 b | 1.31±0.38 a | 5.53 | 1 | 0.024* | 1.53±0.63 | 1.53±0.77 | 1.34±0.95 | 0.85 | 2 | 0.434 |
