# Supplementary material for: The effects of mycorrhizal colonization on phytophagous insects and their natural enemies in soybean fields
Source: PLoS One. 2021 Sep 22;16(9):e0257712. doi: 10.1371/journal.pone.0257712 (PMC8457447; doi:10.1371/journal.pone.0257712)
Supplement: S4 Table — Values represent correlation coefficients. **: P <0.05; *: P<0.1. (DOCX) [file pone.0257712.s004.docx]

**S4 Table.** Correlation between the rate of arbuscular mycorrhizal fungi colonization in the roots of soybean and the abundance and richness of insects at Varennes and Saint-Simon. Values represent correlation coefficients. ^**^: *P* <0.05; ^*^: P<0.1

| **Site** | **Variables** | **Kendall’s *tau***  **coefficient** | ***P*** |  |
| --- | --- | --- | --- | --- |
|  |  |  |  |  |
| **Varennes** | Piercing-sucking insects | -0.246 | 0.016** |  |
|  | Chewing insects | -0.204 | 0.052* |  |
|  | Aphid natural enemies | -0.114 | 0.449 |  |
|  | Aphids without *A. glycines* | -0.031 | 0.767 |  |
|  | *Aphis glycines* | -0.179 | 0.103 |  |
|  | *Empoasca* spp. | -0.162 | 0.119 |  |
|  | Shannon index of phytophagous insects | -0.267 | 0.008** |  |
| **Saint-Simon** | Piercing-sucking insects | 0.080 | 0.436 |  |
|  | Chewing insects | 0.075 | 0.500 |  |
|  | Aphids natural enemies | -0.061 | 0.554 |  |
|  | Aphids without *A. glycines* | 0.075 | 0.479 |  |
|  | *Aphis glycines* | 0.002 | 0.985 |  |
|  | *Empoasca* spp. | 0.020 | 0.848 |  |
|  | Shannon index of phytophagous insects | 0.158 | 0.116 |  |
