# Supplementary material for: The effects of mycorrhizal colonization on phytophagous insects and their natural enemies in soybean fields
Source: PLoS One. 2021 Sep 22;16(9):e0257712. doi: 10.1371/journal.pone.0257712 (PMC8457447; doi:10.1371/journal.pone.0257712)
Supplement: Attachment — Submitted filename: Response to Reviewers.docx [file pone.0257712.s006.docx]

**Response to Reviewers, PLOS ONE**

Thank you for your time handling our manuscript. We here address the concerns raised by the reviews of the first version. Reference to specific line numbers here refer to the clean version of the revised manuscript (not the one with track changes). The most important limitation of the manuscript concerned the probable insufficiency of microbial inoculation since there was as much mycorrhizal infection in the control as in the experimental plots. To address this concern, we emphasized the uncertain nature of the proximate cause of the results of the inoculation experiment. That is, we explicitly acknowledge that the inoculation did not increase the level of mycorrhization and that we did not measure the microbial diversity (L215-219) and, therefore, that we can only surmise that these unevaluated factors played a role in (L305-307), for example, increasing the abundance of piercing-sucking insects in the triple-inoculant plots. As a consequence, we recommend that future research evaluate the full biological diversity of the rhizosphere (L20-21 and L373-376). We maintained the results section largely intact but, to refocus it more towards the evaluation of overall mycorrhization and away from the treatment experiment, we transferred former Tables 3 and 4 to the supplementary materiel. Finally, we revised the title of the manuscript to better reflect the content.

We implemented the journal requirements: 1) style requirements; 2) methods section with additional information concern the fields site access (L135-137); 3) funding information that we removed from the Acknowledgments section and added to cover letter.

Here are the responses to the questions of reviewers.

**Reviewer #1:** “there is a big problem: the success of inoculation of bacteria has not been verified and the success of AMF is not so sure, seen that there are not differences in the mycorrhization rate among control, MR and MRB. The authors speak about a possible explanation for this result, but a doubt on AM inoculation failure remains. If the inoculation has not worked, here the authors are seeing the effects of the native population present in the soils on which there are not data. In this case all the hypothesis about inoculation are not valid. Other experiments or changes in the hypotheses to be tested would be necessary.”

- Thank you very much for your helpful criticism. We hope that the changes described in our paragraphs above address the reviewer’s concerns. We agree that the hypotheses regarding the inoculation treatments are not fully supported given that 1) the treatments did not visibly affect the rate of mycorrhization and 2) we did not measure the relative diversity of the microbial rhizosphere. We also agree that a better comprehension of the microbial rhizosphere in general and, especially in the context of the hypotheses we proposed, how it is affected by soil inocula, would help evaluate their effects on the multitrophic system we studied. However, even microbiologists admit to a lack of understanding of the microbial diversity of the rhizosphere and of the difficulties of studying it (Hepper et al. 1988; Dean et al. 2009; Engelmoer et al. 2014) and, unfortunately, we are not in a position to retrospectively evaluate the very complex microbial flora of our study system. Nonetheless, we think our limited results are worthy of being made available to interested readers because we did in fact detect an effect of inoculation, even if we cannot explain its proximate cause. We hope our experience will help inform future research.

**Major comments**

**Introduction**
At line 24 there is a mistake. Glomeromycota is not a phylum but a subphylum on the base of Spatafora et al. 2016.

- L28: indeed, Schüßler et al. (2001) introduced the phylum *Glomeromycota* to accommodate arbuscular mycorrhizal fungi. But Spatafora et al. 2016 introduced subphylum *Glomeromycotina* Spatafora & Stajich (demoted the phylum to subphylum) and accepted under phylum Mucoromycota. However, certain authors such as Tedersoo et al. 2018, Wijayawardene et al. 2018, maintained the phylum *Glomeromycota*.

**Material and methods
Why do the authors select these two sites with different sizes?**

- L97-98: the experiment was done in collaboration with a company producing and promoting biofertilizers that also provided the living materiel. The sites and experimental design had been already established by the company (L135-137).

**Could the inoculation dose explain the results of inoculation? Are there proofs about the success of this dosage in similar fields and with the same plant?**

- Thank for your questions regarding inoculation. Indeed, the effects of plants inoculation with commercial strains in natural environment remains a challenge. Because in natural conditions, many of these organisms exist indigenously and the interaction with the plant depend on several factors: the quantity and viability of the strains in the soil, the agricultural practices that are unfavorable to the beneficial organisms, the soil, and climatic conditions.
- In L304-312 we explained this situation with the case of AMF because in the control plots, we had colonization independent of inoculation. This confirms that the soil microbiome can play an important role in the microorganism-plant relationships. You were right about the worries raised concern the attribution of the effects due to inoculation. But not having evaluated the mycorrhizal and bacterial component of the rhizosphere, we can make assumptions to explain the results. What we should focus more is the core of this study which remains the correlation between mycorrhizal infection and insect abundance and richness.
- We are not aware of any published studies that have tested the relative success of various dosages in similar soybean fields.

**The authors speak about inoculation, but they find AMF in control….so it is not inoculation the right term. In the figure they speak about colonization that is more suitable. I suggest modifying the terminology in the manuscript.**

- We reserved the term "inoculation" to refer to the commercial strains put into the soil at the time of seeding. Thank for your suggestion.

**I am not so sure that OTUs can be used when there is not molecular identification but only morphological. The current definition is this: the term "OTU" is also used in a different context and refers to clusters of (uncultivated or unknown) organisms, grouped by DNA sequence similarity of a specific taxonomic marker gene. It is difficult for me to understand the OTUs not in relation to DNA and level of insect identification.**

- L168-171: you are right about the term “OTU" in a microbial context, but in entomology, operational taxonomic units (OTUs) can be used to indicate categories of specimens that share morphological characters: these are sometimes call morphospecies. We cited Favret et al 2019 as reference.

**Results**
**At lines 219-220 add the reference to data or table or supplementary or data not shown. With the doubt about the success of inoculation all the results based on this are doubtful (3.1 and 3.2 paragraphs).**

- L213 and L225 respectively 3.1 and 3.2 paragraphs: as explain above, it is difficult to assert that inoculation did not work, even if it was not measurable, due to the certain presence of indigenous mycorrhizal spores. Of course, this is the difficulty of working in a natural setting instead of the controlled laboratory, but even if we cannot be definitive, we are still left to explain why the plots with triple-inoculant-treatments saw an increase in the number of piercing-sucking insects.
- L304-312: we explain these results by a possible influence of each or both inoculants (introduced and native). For example, a study by Dean et al. 2009 (<https://doi.org/10.1007/s11104-009-9924-1>) compared 2 sources of *Bradyrhizobium japonicum* (commercial and native) on soybean aphid and showed a decrease in the aphid abundance with the native strain. Without a genetic analysis to get the identity of the strains, there could think that this effect was due to the introduced inoculants.
- Because of the presence of native strains in our study and not having measured the microbial community in the soil, we think that it is hard to reject these results.

**Discussion**
**At lines 318-319 the sentence is not right: the effects are not on treatments, but on variables.**

- L289-292: sentence changed. The term "treatments" referred to inoculant treatment with the 3 groups (control, MR and MRB) (S1 Table).

**In the discussion it is useful to put off to tables or figures.**

- L288-358: done.

**At lines 350-352 authors speak about the effects of bacteria but they have never checked the inoculation success and persistence.**

- L310-312: sentence changed.

**At line 424 they speak about high, but it is very difficult to say this, seen that there are not differences among the treatments.**

- L358: sentence removed; here, it is to specify that despite a measurable effect of inoculation, there was yet an important level of mycorrhizal colonization in roots (L308-310). Term changed.

**Table
In Table 1 Piercing-sucking insects subtotal 20: which numbers do produce 20?**

- L184 (Table 1): 20 is the sum of OTUs =13+1+6 respectively Aphids subtotal+*Empoasca* spp.+ Other piercing-sucking insects.

**Figures
I think that Fig 2, 3 and 4 are not necessary, seen that there is information in Table 4.**

- We kept the figures to show the direction of the correlation and put the Table 4 as supplementary material (S4 Table).

**Minor comments** (the responses are in bold)

At line 39 replace “dependent on” with “related to”- **L40: word changed.**

At line 46 move “simultaneously” after can- **L47: word removed.**

At line 53 add “s” at the verb- **L52: letter added.**

At line 55 replace “One of” with “among” and add “there” before are- **L54: sentence changed after revision.**

At line 59 replace “reducing” with “limiting”- **L58: word replaced.**

At line 63 replace “quality” with “feature”- **L61: word replaced.**

At line 67 move the adverbs before affect and eliminate “be it”- **L64: sentence modified**.

At line 70 add “also” after can- **L68: word added**.

At line 82 add “a” after of – **L79: “a” added**.

Lines from 114-116 replace “high and low” with maximum and minimum - **L109-111: words replaced**.

Check along the text not to separate subject and verb with comma: **done**
At line 134 move “also” after the verb- **L129: word moved.**

At line 141 add “insects” after sampled- **L139: word added**.

At line 149 replace “to” with “in” and move the adverb after was- **L147: words replaced and moved.**

At lines 177-178 remove species and genus and replace “these” with “this”- **L174-175: words changed.**

At line 181 eliminate “measures of”- **L178: words removed**.

At line 197 add “were studied using” and eliminate “, we used”- **L196-197: sentence changed after revision.**

At line 219 add “e” for here- **L212: sentence removed after revision.**

Move the comments in the results and put in the right section (lines 265-266; 282-283 moreover it is a sentence non clear)- **L240-241: sentences changed.**

At line 292 add “insects” after chewing- **L270: word added.**

At line 323 replace “we expected” with we can explain- **L294: sentences changed.**

At line 324 replace “may be explained by” with “with”- **L295: sentences changed.**

Chose as to write above-ground or aboveground- **L294-295: suggestion done**.

At line 339-340 rephrase and add reference to table- **L308-309: sentence changed.**

At line 349 replace “in high abundance” with abundantly- **L310: sentence changed**.
At line 358 replace “most” with “majority the”- **L292-293: sentence changed.**

At line 360 replace “relative” with compared- **L323: sentence changed.**

At line 376 replace “that” with i.e.,- **L328: word replaced.**

At line 430 there is some mistakes-**L362: sentence corrected.**

**Reviewer #2:**  Major drawbacks are the results of mycorrhizal colonization following inoculation with commercial inoculants, as they were similar with those obtained in control theses. Authors should revise the whole manuscript taking into account this issue, avoiding conclusive or deductive sentences on the effects due to inoculants (i. e. L267-268 change with something like “a significant higher number of insects was collected…” instead of “…the inoculants did affect abundance”). In this perspective, the correlation between insect abundance/diversity and mycorrhizal colonization rate (irrespective of the treatments) becomes therefore the main core of the manuscript. My suggestion is to shorten the title in “The effects of mycorrhizal infection on phytophagous insects in soybean fields”. From this point of view, discussion (especially the first part L318-331) needs to be reformulated, also because here and there it is a repetition of results.

In conclusion, taken together your data seem to indicate that higher abundance of pierce-sucking insects is observed with low mycorrhizal colonization rate, and also in the treatment MRB (not MR), at least in one site (table 3). A hypothesis could be that increase in insect abundance is due more to the plant growth-promoting bacterium, Bacillus pumilus, rather than the AMF. This might be discussed, with appropriate literature support.

- Thank you for the kind words regarding the manuscript (not repeated here) and especially for the suggestions for its improvement. We have addressed the major criticism regarding the unimproved rate of mycorrhization in the experimental as opposed to the control plots in the replies to the editor and reviewer 1. Indeed, the introduction and especially the discussion were significantly modified.

**Title of the manuscript**

- L1-2: as suggested, we revised the title to emphasize the issue of mycorrhization correlated with the abundance and richness of phytophagous insects, which is the main core of the manuscript.

**The whole manuscript**

- Indeed, we noted that the question of how inoculations work is not explicit, and sometimes we went very quickly to certain conclusions, which was often confusing. In the revised manuscript, we provided more details about inoculations (introduction of commercial strains into the soil, which have been selected for their ability to enhance crop yields) and inoculants, which may be indigenous strains present in the soil in addition to the introduced strains.

**Results**

**L267-268**

- As you suggested, we revised the manuscript by presenting our results with appropriate terms by avoiding deductive or conclusive sentences (i.e. L242-243).

**Discussion**

**L318-331**

- The first part of the discussion is reformulated considering the title and the results (L288-311).

**Taken together the data seem to indicate that higher abundance of pierce-sucking insects is observed with low mycorrhizal colonization rate, and also in the treatment MRB (not MR), at least in one site (table 3). A hypothesis could be that increase in insect abundance is due more to the plant growth-promoting bacterium, *Bacillus pumilus*, rather than the AMF.**

- L303-309: we agree with the reviewer that the bacterial components of the rhizosphere may have affected the insect populations. In light of this, we recommend further work be done to evaluate the taxonomic make-up of the microbial rhizosphere and its effects on the plants and higher trophic levels (L19-21).

**Minor comments (**the responses are in bold**)**

L24: Glomeromycota in italics-**L28: changed**

L35: Delete “Meanwhile” and the comma between the subject “free-living PGPR” and the verb “can promote”- **L39: meanwhile and the comma removed.**

L55-74: make a unique paragraph-**L54-72: done**

L184: avoid “versus”, there is no an opposition, only a distinction in different categories-**L181: “versus” delete and replace by “and”.**

L216: add “measured in different plots” after “colonization rate. It should improve clarity of the analysis- **L211:** **sentence modified as you suggested.**

L219: here not her-**L212: sentence removed after revision**
L219-224: Maybe the passive form for these sentences is more appropriate- L212: **sentence changed.**

L219-220 and L224-225: these sentences are repeating the same concept-**L215-216: sentence removed and corrected.**

L248: the data were pooled irrespective of K+/K- treatments, isn’t it? Please specify here and in caption to Table 3-**L233-234:** y**es, precision done and in caption to S1 Table.**

L290: add “irrespective of treatments” after “rate of mycorrhization”. It should improve clarity of the analysis- **L268**: **sentence modified as you suggested.**

L358-359: please rephrase, not very clear. Which “other effects”?- **L291-292: sentence changed**.

L370-371: use “similar” instead of “the same”, Empoasca spp. are known as many mesophyll feeders though not exclusive, aphids mainly phloem feeders-**L323: sentence removed after revision.**
