# Supplementary material for: The effects of mycorrhizal colonization on phytophagous insects and their natural enemies in soybean fields
Source: PLoS One. 2021 Sep 22;16(9):e0257712. doi: 10.1371/journal.pone.0257712 (PMC8457447; doi:10.1371/journal.pone.0257712)
Supplement: Attachment — Submitted filename: Response to Reviewers.docx [file pone.0257712.s007.docx]

**Response to Reviewers, PLOS ONE**

Thank you for your time handling our manuscript. We hereby confirm that the reference list is update, complete and correct. Any reference cited in the text is in the bibliography.

Here is the response to the question of reviewer 2.

**Minor issue: (the response is in bold)**

L219- Change “microbial community is general” with “microbial community in general”- **L219:** **word replace in the sentence**
