# Supplementary material for: The effects of mycorrhizal colonization on phytophagous insects and their natural enemies in soybean fields
Source: PLoS One. 2021 Sep 22;16(9):e0257712. doi: 10.1371/journal.pone.0257712 (PMC8457447; doi:10.1371/journal.pone.0257712)
Supplement: Attachment — Submitted filename: Response to Reviewers.docx [file pone.0257712.s008.docx]

**Response to Reviewers, PLOS ONE**

Thank you for your time handling our manuscript. We here address the concerns raised by the reviews of the academic editor. Indeed, you were right about the statistics and thank you for your pertinent observations. We confirm that we performed an interaction between microbial treatments and potassium. Certainly, the way we presented the results could lead to believe that we had not performed it. Also, the lack of some details in our explanations may not allow for a better understanding. Thus, we revised the results tables with more clarity (L224-Table 2 and S Tables) and made a careful reading of this revised version of the manuscript. Reference to specific line numbers here refer to the clean version of the revised manuscript (not the one with track changes).

Here are the responses to the comments and questions raised by the academic editor:

**1-You mention 6 treatments, but your analysis suggests a factorial design: C, MR and MRB crossed with K+ and K-.**

L115-120-Indeed, it is a factorial design with 2 factors: an inoculant factor with 3 levels (C, MR and MRB) crossed with another factor, the potassium with 2 levels (K+ and K-); which gives 3x2 or 6 plots/bloc replicates 8 times.

**2-Did you test for an interaction between K and microbial treatments? Why not?**

Yes, we tested the interaction between microbial treatments and potassium. Maybe we were not precise, but the interaction was tested. Of all tests, only the interaction was observed with *Aphis glycines* at Saint-Simon (S Tables). That motivated us to make 2 tables (S1 effects of inoculants on insects and S2 effects of potassium). But in the revised manuscript we merged the 2 tables. Below figure is an example on the output of analysis of variance apply to the Linear mixed model (LMM) when testing the effects of inoculants and potassium on piercing-sucking insects at Varennes. It shows individual and the interaction effects between the two factors (inoculants and potassium).

**aoVar_piq** : LMM model

aovVar_piq<-lmer(Tot_piq~Trait*K+(1|Bloc)+(1|Bloc:Trait)+(1|Bloc:K), data=Analys_Var)

**Trait**: inoculants treatments

**K**: potassium

**Trait:K**= interaction

**3-Why did you used a mixed model (i.e. what factors are random and what factors are fixed)? Where is the block effect tested? Please report all stats (i.e. K treatment and block effects) for all tests; please also report df (missing in some tests).**

L200-201: we used mixed model because of fixed effects (inoculants treatments, potassium) and random effects (blocks). The block effects are included in the model formulation:

Model<-lmer (variable~Trait*K+(1|Bloc) +(1|Bloc: Trait) +(1|Bloc: K), data=Data)

Trait=Inoculants	K=Potassium

**4-It is unclear how some of the treatment comparisons were done. For example, in Lines 242-247, did you do comparisons of microbial treatments separately for the K+ and K- treatments? The df of 2 and 21 (L242) and 2 and 14 (L247) of the tests suggest that; please provide a more explicit description of the tests performed and the results obtained.**

In this paragraph, we would like to provide more details, but the explanations not sounded. Otherwise, there was a significant interaction between microbial treatments and potassium for the soybean aphid, *Aphis glycines* (F2.21=6.69, P=0.006, Fig 1; S1-S3 Tables). Later, we went into more details by showing the behavior of the microbial inoculants in the presence or absence of potassium, which effectively makes a separate analysis. In the revised manuscript, we corrected all the paragraph (L245-254).

Here are the responses to the minor issues raised by academic editor and reviewer (s).

**5-Minor changes: (the response is in bold)**

L73 change “AM fungi” to “AMF” for consistency-**L73: word change**
L79: delete “in an agronomic system,” (sentence is too long, and at the end you mention soybean an under field conditions…)-**L79: sentence deleted**

**L82-L100: number of rhizobium bacterium strain corrected**.

L84: replace “is” by “are”-**L84: word replaced.**

L123/129: fix typo in “block” (check for others I may have missed…)-**L123/129: word corrected and in the whole manuscript.**

L198: the reference provided for the Shannon diversity index equation uses ln, not log2; please provide a reference using the formula you stated-**L199: you are right, it was an error and we corrected it.**

L201: change “responding variables” to “response variables”-**L203: sentence changed**
L207: rewrite “In a case of significance difference was observed, “to “When significant differences were observed,”-**L208-209: sentence rewritten.**

L211: please justify the use of Kendall correlation coefficients-**L211**: **We used Kendall correlation because our data did not meet all the assumptions of normal distribution to apply Pearson correlation that we made first.**

L292: change “excepted” by “except for”- **L299: word changed.**

L341: change to “On the other hand and in line with our investigation,”-**L348: sentence changed**
L346-348: change to “Therefore, it is possible that the negative correlation between
chewing insects and AMF plant colonization observed in our study is due to the generalist feeding habits of the chewing insects sampled”-**L353-354: sentence changed.**

**6-Please revise all citations, several are incomplete, including: 12, 23, 24, 25, 38, 48, 58. Please follow journal format regarding the provision of doi (several are missing)**

We hereby confirm that the reference list is update, complete and correct. Any reference cited in the manuscript is in the bibliography.

**L426**: citation **12**- is a book-replaced by another citation also pertinent

**L463**: citation **23**- there is no doi associated with this citation on the journal site; but it has been completed by adding the journal name, the volume number, the year and the pages number (suggested citation)

**L466**: citation **24**: there is no doi associated with this citation on the journal site; but it has been completed by adding the journal name, the volume number, the year and the pages number (suggested citation)

**L469**: citation **25-**citation corrected

**L515**: citation **38**- became 39- corrected

**L549**: citation **48**-became 49- corrected

**L579**: citation **58-**became **59-**corrected
