# Supplementary material for: The effects of mycorrhizal colonization on phytophagous insects and their natural enemies in soybean fields
Source: PLoS One. 2021 Sep 22;16(9):e0257712. doi: 10.1371/journal.pone.0257712 (PMC8457447; doi:10.1371/journal.pone.0257712)
Supplement: Attachment — Submitted filename: Response to Reviewers.docx [file pone.0257712.s009.docx]

**Response to Reviewers, PLOS ONE**

We thank you for your important comments and suggestions. We here address the concerns raised by the reviews of the academic editor. Reference to specific line numbers here refer to the clean version of the revised manuscript (not the one with track changes).

Here are the responses to the comments and questions raised by the academic editor:

1- **Section 3.1. There are no Anova test results reported for the main effects,**

**Potassium and inoculation treatments, and their interaction. Please report them in the text. In Table 2 you report tests comparing the inoculant treatments (C, MR, and MRB) separately within each K treatment. This is justified if you have a significant interaction between inoculant and K treatments, however that was not indicated. I think is ok to present the means+/- SE of the 6 treatments, as you have them currently in Table 2, but not the anova tests within factors unless you have significant interactions (in which case you also need to check differences for K within each inoculation treatment). Also, always report both degrees of freedom for your anova tests, numerator and denominator, separated by commas in your tables or as subscripts after the F in the text.**

**Response:** L216-229: Section 3.1. We added a new table named S1 Table (Supplementary materiel) with the Anova test results for the main effects, potassium, inoculation treatments, and their interaction. We also keep the Table 2 with means because we mentioned it as support in the discussion section (L315). For the degrees of freedom as numerator and denominator with F values, we reported them in all the text.

**2. Supplementary tables: do not repeat values in tables S1 and S2. I suggest you delete the stats in table S2 (already reported in S1) and only leave the different letters indicating the Tukey test for pierce-sucking insects at Varennes and for the main effect test on Empoasca in Saint-Simon. I suggest you delete S3, as the same information is presented in Figure 2.**

**Response-** With the insertion of the new S1 table, the former Tables S1 and S2 are know Tables S2 and S3 respectively in the revised version. In the new S3 Table, we deleted the stats and left only piercing-sucking insects at Varennes and *Empoasca* spp. at Saint-Simon. We also deleted the former S3 Table as you suggested.

**3-Minor changes: (the response is in bold)**

L 194: replace “normal logarithm” by “natural logarithm”-**L194: word replaced**

L 238: F test number does not match exactly table S1, please check-**L238: F test number changed**

L 296/300/etc.; global change: please change “S1 and S2 Tables” to “Tables S1 and S2”-**L295-300: words changed**

L 310: replace “fields conditions” by “field conditions”-**L309: words replaced**

L313: to avoid repetition with the previous sentence, I would replace “In field conditions,” by “Further,”-**L312: sentence replaced by the suggested one**

L357: replace insects’, by “insect”-**L356: word replaced**

L374: delete “the” before mycorrhizal-**L373: word deleted**

L375: delete “can” before depend-**L374: word deleted**

L385 (**You mean L378? because the L385 is in the section of Acknowledgements)**: after “in our study,” add “suggest that”-**L377: sentence added**
